# Nursing-led childbirth preparedness sessions: enhancing self-efficacy and maternal outcomes among primiparous women

**DOI:** 10.1186/s12912-026-04404-6

**Published:** 2026-03-02

**Authors:** Huda Hamdy Mohammed, Azza Ali Abd El Hamed, Nagwa Abd-El Fadeel Afefy, Nadine Alaa Sherif, Sahar Mansour Ibrahim

**Affiliations:** 1 Department of Maternal & Newborn Health Nursing, Faculty of Nursing, Cairo University, Cairo, Egypt; 2 Department of Obstetrics & Gynaecology, Faculty of Medicine, Cairo University, Cairo, Egypt

**Keywords:** Childbirth preparedness, Childbirth self-efficacy, Primiparous women, Labor pain, Anxiety, Labor duration, Nursing intervention

## Abstract

**Background:**

Primiparous women commonly experience heightened anxiety and low childbirth self-efficacy, which may intensify pain, prolong labor, and increase obstetric interventions. Nurse-led childbirth preparedness may strengthen coping confidence and improve intrapartum outcomes.

**Aim:**

To examine the effect of nursing-led childbirth preparedness sessions on childbirth self-efficacy, anxiety, pain intensity, labor duration, and mode of delivery among primiparous women.

**Methods:**

A quasi-experimental post-test-only non-equivalent control group study was conducted at Kasr Al-Ainy University Hospital, Cairo, Egypt. A purposive sample of 140 low-risk primiparous women was allocated to an intervention group (*n* = 70) and a control group (*n* = 70). The intervention comprised two structured nurse-led group sessions (≈45 minutes each) delivered at 32–34 weeks’ gestation, a supportive Arabic booklet, and weekly follow-up phone calls. Outcomes were measured during labor using the Arabic Childbirth Self-Efficacy Inventory (ACSEI), the Anxiety Assessment Scale for Pregnant Women in Labor (AASPWL), and the Numerical Rating Scale (NRS) for pain, with labor duration and mode of delivery extracted from clinical records. Group comparisons were performed using independent *t*-tests and chi-square tests.

**Results:**

Women who received the preparedness sessions demonstrated significantly higher total childbirth self-efficacy than controls (ACSEI: 226.07 ± 7.12 vs 200.25 ± 5.96; *t* = 23.27, *p* < 0.001). Anxiety levels differed significantly, with a higher proportion of low anxiety in the intervention group (51.4% vs 22.9%; χ^2^ = 14.53, *p* = 0.002). Mean pain scores were lower in the intervention group during latent (4.66 ± 0.68 vs 4.96 ± 0.82; *p* = 0.020) and active acceleration phases (6.07 ± 0.80 vs 7.14 ± 0.98; *p* < 0.001). First-stage labor duration was shorter in the intervention group (8.85 ± 1.89 vs 10.17 ± 1.94 hours; *p* < 0.001), as was second-stage duration (63.9 ± 22.6 vs 89.6 ± 26.3 minutes; *p* < 0.001). Mode of delivery also differed significantly (χ^2^ = 7.24, *p* = 0.027), with lower cesarean section rates in the intervention group (17.1% vs 34.3%).

**Conclusion:**

Nursing-led childbirth preparedness sessions were associated with higher childbirth self-efficacy and lower anxiety and pain, as well as shorter labor duration and fewer cesarean births among primiparous women. Given the quasi-experimental design, results should be interpreted as associations and warrant confirmation in randomized multi-center studies.

**Trial registration:**

Not applicable (quasi-experimental, non-randomized study).

## Introduction

Pregnancy and childbirth encompass a protracted process, marked by physiological, psychological, and emotional transformations that can yield either favorable or unfavorable consequences for the woman’s well-being and the family unit. About 15% of Primiparous women express fear or anxiety about what will happen in labor [1]. Primiparous women often face challenges such as misunderstandings about childbirth, unanswered questions, and inadequate information, with up to 70% experiencing high levels of anxiety, stress and lack of self-efficacy due to a lack of knowledge [2].

Low self-efficacy led to heightened anxiety and stress, causing the release of stress hormones such as adrenaline, noradrenaline, and cortisol from the adrenal gland. These hormones increase heart rate; blood pressure; reduce uterine blood flow; disrupt contraction patterns; affects placental function; decrease pain tolerance and increase muscle tension, leading to prolonged labor, a greater likelihood of medical interventions like epidurals, assisted birth and increasing incidence of caesarean section and a less positive childbirth experience overall [3]. A woman’s self-efficacy plays an important role in how she experiences childbirth. Primiparous women, who are more assured in their capacity to control their labor experience better labor, suffer less pain, and use analgesia less frequently [4].

Self-efficacy is defined as a dynamic cognitive process that explains a personal belief to perform a required behavior in each situation successfully. It is important marker for behavior change, self-control, and primiparous women’s coping abilities during labor [5]. The woman’s self-efficacy for labor has a major role in her ability to use coping mechanisms during labor. A woman’s high self-efficacy plays an important role in how she experiences childbirth. Primiparous women, who are more assured in their capacity to control their labor experience better labor, suffer less pain, and use analgesia less frequently [6].

Self-efficacy is influenced by individuals’ past experiences in mastering the situation at hand, the experiences of others, knowledge, verbal persuasion, and degree of emotional and physiological arousal [7]. It is composed of two parts, outcome expectancy and self-efficacy expectancy. Outcome expectancy refers to trusting that behavior will lead to a certain outcome. Self-efficacy expectancy refers to the individual’s belief that they can perform that behavior successfully to produce the desired outcome [8]. Enhancing childbirth self-efficacy, Primiparous women needs to be prepared for childbirth preparation.

Childbirth preparedness sessions initially began in the early 1900s to encourage pregnant Primiparous women to come for antenatal care, plays a significant role in the physical and psychosocial preparedness of readying for both Primiparous women and those expecting subsequent children after several years [9, 10]. By attending these sessions pregnant Primiparous women will be equipped with essential knowledge, skills, and emotional support, including insights into labor stages, learn about different situations during labor, pain relief techniques and newborn care [11].

Childbirth preparedness sessions significantly benefit both maternal and fetal outcomes by improving the mother’s physical, emotional, and psychological readiness for labor. For first-time mothers, these sessions help reduce anxiety, increase confidence, and teach effective pain management techniques, which often result in shorter labor durations and fewer medical interventions, such as epidurals or cesarean sections [12]. According to Olza, Leahy-Warren, Benyamini, Kazmierczak, Karlsdottir, Spyridou, et al., (2018), childbirth preparedness sessions play a vital role in enhancing self-efficacy, fostering emotional readiness, and reducing fear, which collectively improve labor outcomes [1].

Also, Bandura, self-efficacy relies on social and self-regulatory skills, self-belief, and mastery of new abilities through information, skill development, and social support [13]. Childbirth preparedness aligns with these elements by enhancing self-efficacy in Primiparous women, helping them manage labor more confidently and reduce reliance on medical interventions [14]. Recent study done by, Abd El-Kader (2024) evaluated the effectiveness of childbirth self-efficacy enhancing classes on labor length and outcomes among Egyptian Primiparous women [15]. Results documented that, approximately 68.9% of Primiparous women in the experimental group gave birth vaginally, compared to 29.7% of those in the control group with statistically significance differences. In the experimental group, labor took an average of 8 to 12 h, but it took more than 12 h in the control group. The study concluded that, antenatal education classes have a real chance of assisting Primiparous women in increasing their childbirth self-efficacy and improving maternal and neonatal outcomes.

A systematic review done by Alizadeh-Dibazari, Maghalain, & Mirghafourvand, (2024) examines the impact of childbirth preparation sessions on both psychological and physical maternal outcomes [16, 17]. The findings highlight that these sessions significantly reduce anxiety, fear, and stress during labor, enhancing self-efficacy, improved pain management and higher maternal satisfaction. Physically, Primiparous women attending these sessions are less likely to require medical interventions like cesarean sections and epidurals, resulting in a more natural birth process and better overall maternal outcomes [18].

Despite existing studies, there are not enough studies that examine the effect of childbirth preparedness on childbirth self-efficacy among Primiparous women, particularly in Egypt. This gap leaves an incomplete understanding of how these sessions specifically enhance childbirth self-efficacy for primiparous women. So, this study aimed to fill this gap by examining the effect of a nursing-led childbirth preparedness sessions on self-efficacy, and maternal outcomes among Primiparous women.

## Conceptual framework

This study is guided by Bandura’s self-efficacy theory, which proposes that confidence in coping is strengthened through (1) mastery experiences, (2) vicarious learning, (3) verbal persuasion, and (4) management of physiological/emotional arousal. The childbirth preparedness intervention was designed to operationalize these mechanisms: skill practice and rehearsal (mastery), group discussion and shared experiences (vicarious learning), nurse-led encouragement and feedback (verbal persuasion), and breathing/relaxation strategies to reduce fear–tension responses (physiological regulation). We therefore hypothesized that stronger childbirth self-efficacy would be associated with lower intrapartum anxiety and pain, shorter labor duration, and more favorable delivery outcomes.

## Aim of the study

The present study aimed to examine the effect of nursing-led childbirth preparedness sessions on childbirth self-efficacy, anxiety, pain intensity, labor duration, and mode of delivery among primiparous women.

## Operational definitions

For the current study, the following operational definition addressed:

**Childbirth Preparedness:** in this study means antenatal preparation sessions offered to Primiparous women which will equipped Primiparous women with essential knowledge, practical skills, and emotional support. Implemented through sessions and educational booklet.

**Childbirth Self-Efficacy:** in the current study means that the primiparous woman’s belief in her ability to manage childbirth as measured by Childbirth Self-Efficacy Inventory (CBSEI).

**Maternal Outcomes:** in the current study means maternal anxiety level, pain level and labor duration as measured by Anxiety Assessment Scale, Numerical Pain Rating Scale and labor assessment.

## Research hypotheses

To achieve the stated aim, the following hypotheses were formulated:

### H1

Primiparous women who attend a childbirth preparedness session will have a higher level of self-efficacy during labor than those who don’t attend a childbirth preparedness session

### H2

Primiparous women who attend a childbirth preparedness session will report lower anxiety level than those who don’t attend a childbirth preparedness session

### H3

Primiparous women who attend a childbirth preparedness session will report lower labor pain level than those who don’t attend a childbirth preparedness session

### H4

Primiparous women who attend a childbirth preparedness session will have shorter labor duration than those who don’t attend a childbirth preparedness session

## Methods

### Study design

A quasi-experimental post-test-only non-equivalent control group design was selected because random allocation was not feasible within the routine workflow of the study setting. This design is commonly used in real-world maternal health service evaluations where individual randomization may be operationally challenging. Both groups were assessed post-intervention and findings interpreted as associations between primiparous women in childbirth self-efficacy and maternal outcomes rather than definitive causal effects.

### Setting

The study was conducted at Kasr Al-Ainy University Hospital, affiliated with Cairo University in Egypt. Recruitment and childbirth preparedness sessions took place in the women’s care clinic, which provides free antenatal and gynecological services and serves approximately 33,000 women annually. The clinic includes dedicated antenatal follow-up rooms, laboratory services, ultrasound examination rooms, and waiting areas, and is staffed by obstetricians, gynecologists, and diploma nurses. Follow-up and outcome assessments were conducted in the labor unit (Section 10) of the same hospital, which includes two admission rooms, nine first-stage labor beds, and three fully equipped delivery rooms, with an average of 1000 monthly deliveries (Statistics Department, 2024). This high case load ensured adequate access to eligible Primiparous women.

### Sample

The study targeted Primiparous women attending the women’s care clinic at Al-Kasr Al-Ainy University Hospital, Cairo, Egypt. A purposive sampling technique was employed to recruit Primiparous women who met the inclusion criteria: age 18–35 years, 32–34 weeks of gestation, singleton low-risk pregnancy, ability to read and write Arabic, and no prior attendance at childbirth preparedness sessions. Primiparous women with high-risk pregnancies (e.g., preeclampsia, gestational diabetes, placenta previa), multiple gestations, or previous exposure to structured antenatal education were excluded to reduce confounding variables.

Sample size was calculated using the formula for comparing two independent means, with 95% confidence, 80% power, and an effect size of 0.5. The required number was 63 Primiparous women per group; however, to compensate for a possible 10% attrition, 70 Primiparous women were recruited for both the intervention and control groups, yielding a total of 140 Primiparous women.

$$n = {{2 \cdot {{\left( {{Z_{{\alpha \mathord{\left/ {\vphantom {\alpha 2}} \right. \kern-\nulldelimiterspace} 2}}} + {Z_\beta }} \right)}^2} \cdot {\sigma ^2}} \over {{{\left( d \right)}^2}}}$$

Recruitment occurred in two phases. Primiparous women in the study group were approached at the antenatal clinic, screened for eligibility, consented, and enrolled in childbirth preparedness sessions. The control group was recruited from eligible primiparous women admitted to the labor unit, where they received routine antenatal care. This approach minimized contamination between groups while ensuring comparability.

The purposive sampling strategy, strict eligibility criteria, and adequate sample size strengthened the study’s methodological rigor, ensuring reliable evaluation of the effect of nursing-led childbirth preparedness on self-efficacy, anxiety, pain intensity, and maternal outcomes among primiparous women.

Because the intervention group was recruited antenatally and the control group at labor admission, there is potential for selection or timing bias. To mitigate this risk, eligibility criteria were identical for both groups (age range, gestational age window, low-risk singleton pregnancy, and no prior attendance at childbirth classes), and both groups delivered in the same labor unit under the same clinical protocols. Baseline comparability was assessed using demographic characteristics (Table 1), which demonstrated no statistically significant differences between groups. However, we acknowledge that baseline equivalence on psychosocial variables (e.g., self-efficacy, childbirth fear, pain expectations) could not be fully ensured in this design and is therefore addressed explicitly as a limitation.

**Table 1:** Distribution of the primiparous women in both groups according to their demographic data

Characteristic	Category	Study (n = 70)	Control (n = 70)	χ^2^	p-value
Maternal age (years)	18–	38 (54.3%)	40 (57.1%)	0.14	0.78
	23–	24 (34.3%)	22 (31.4%)		
	≥26	8 (11.4%)	8 (11.4%)		
Education level	Primary	24 (34.3%)	23 (32.9%)	0.30	0.768
	Preparatory	25 (35.7%)	27 (38.6%)		
	Diploma	18 (25.7%)	18 (25.7%)		
	University	3 (4.3%)	2 (2.9%)		
Occupation	Working	10 (14.3%)	14 (20.0%)	0.80	0.502
	Housewife	60 (85.7%)	56 (80.0%)		
Residence	Rural	36 (51.4%)	32 (45.7%)	0.24	0.612
	Urban	34 (48.6%)	38 (54.3%)		
Monthly income	Adequate	20 (28.6%)	22 (31.4%)	0.14	0.854
	In-adequate	50 (71.4%)	48 (68.6%)		

### Data collection tools

Four tools were used in this study to assess outcomes:

1. **Structured Interviewing Questionnaire.** This tool was developed by the investigator after an extensive review of current maternity nursing and obstetric literature. It consisted of five main parts: (1) demographic data (age, education, occupation, residence, and income adequacy); (2) anthropometric measurements (height, weight, and body mass index); (3) obstetric and pregnancy history; (4) current pregnancy details, including gravidity, gestational age, last menstrual period, expected date of delivery, and antenatal follow-up; finally (5) maternal assessment, documenting events and outcomes during the first and second stages of labor, such as prolonged labor, instrumental delivery, or cesarean section.
  The tool was reviewed for content validity by a panel of three experts in maternity and obstetric nursing, who evaluated each item for clarity, relevance, and comprehensiveness. The content validity index (CVI) was 0.91, indicating excellent agreement among experts. Minor revisions were made to linguistic phrasing and sequencing of questions to enhance clarity and logical flow. Test–retest reliability was evaluated among a pilot sample of 15 Primiparous women with a two-week interval, yielding a correlation coefficient of 0.88, confirming the tool’s stability over time.
2. **Arabic Childbirth Self-Efficacy Inventory (ACSEI).** The ACSEI was adapted from the original Childbirth Self-Efficacy Inventory (CBSEI) developed by Lowe (1993), which measures a woman’s confidence in her ability to manage the demands of labor. It assesses two major domains outcome expectancy (belief in the effectiveness of coping behaviors) and self-efficacy expectancy (confidence in one’s ability to perform coping behaviors) across both the first and second stages of labor. The instrument includes 62 items rated on a 10-point Likert scale (1 = not at all confident to 10 = completely confident). Higher scores indicate stronger self-efficacy, with total scores categorized as low (31–103), moderate (104–206), or high (207–310). The Arabic version used in this study was developed and validated by El Kurdy (2016) through forward–backward translation, cultural adaptation, and psychometric testing among Egyptian Primiparous women. Reported Cronbach’s alpha coefficients were 0.86 for the outcome expectancy subscale and 0.90 for the self-efficacy expectancy subscale, confirming high internal consistency. Construct validity was supported by factor analysis, which demonstrated two distinct yet correlated components consistent with the original CBSEI model. In the current study, additional expert review confirmed its linguistic clarity and cultural relevance. Reliability reassessment among the study’s pilot Primiparous women yielded Cronbach’s alpha = 0.89, supporting the scale’s use in this population.
3. **Anxiety Assessment Scale for Pregnant Primiparous women in Labor (AASPWL).** Developed by Duart et al. (2018), the AASPWL was designed to measure labor-specific anxiety through nine items covering two dimensions: concerns about the birth process and maternal role transition. Each item is scored on a 5-point Likert scale ranging from 1 (“not at all anxious”) to 5 (“extremely anxious”), with higher scores indicating greater anxiety. The total score ranges from 9 to 45 and is classified as low (9–18), moderate (19–27), high (28–36), or severe (37–45). For this study, the AASPWL was translated into Arabic following World Health Organization (WHO) forward–backward translation protocols. bilingual maternity nursing experts independently reviewed the translation for semantic and conceptual equivalence, and the Arabic version underwent pilot testing to ensure readability and cultural relevance. Cronbach’s alpha coefficient was 0.77, indicating acceptable internal consistency. Concurrent validity was established through a moderate positive correlation (*r* = 0.61, *p* < 0.01) with the State-Trait Anxiety Inventory (STAI), confirming its sensitivity in detecting intrapartum anxiety.
4. **Numerical Pain Rating Scale (NRS).** Originally developed by Hartrick, Kovan, and Shapiro in 2003 (5), the NRS is a widely used single-item measure of pain intensity. Primiparous women rate their pain on a scale of 0–10, with 0 representing no pain and 10 the most severe imaginable pain. Scores are interpreted as mild (1–3), moderate (4–6), or severe (7–10). The tool’s validity and reliability are well-established across clinical populations, including Primiparous women in labor. The Arabic version, adapted through standardized translation procedures, demonstrated strong face validity and was easily comprehensible to Primiparous women in this study.

### Ethical consideration

The primary official permission was obtained from the Scientific Research Ethics Committee of the Faculty of Nursing, Cairo University, on 29 January 2025 (No. 8–1-2025). The investigator explained to the primiparous women the purpose, nature, and importance of the study. Informed written consent was obtained from those who were willing to participate after they were assured that their participation was voluntary and that the study posed no risks or hazards to them. Each primiparous woman was also informed that she had the right to withdraw from the study at any time without any effect on the care she received. Confidentiality and anonymity were ensured by coding the data and keeping all collected information in a secure location. The final official permission was obtained from the Scientific Research Ethics Committee of the Faculty of Nursing, Cairo University, after complete data collection.

### Procedure for data collection

Data collection was carried out over several sequential phases to ensure methodological rigor and participant compliance. The process began with the preparation phase, during which the investigator obtained official approval from the hospital administration and coordinated with the Primiparous women’s care clinic and labor unit staff to facilitate recruitment and data collection. Structured tools and the supportive educational booklet were developed based on current evidence and underwent expert review for content validity. Training sessions were also conducted with the assisting nurses to familiarize them with the study protocol, assessment tools, and ethical considerations, thereby ensuring consistency in implementation.

### Recruitment phase

Primiparous women attending the women’s care clinic at Kasr Al-Ainy University Hospital were screened against the inclusion and exclusion criteria. Eligible Primiparous women were approached in the antenatal clinic waiting area and provided with a clear explanation of the study’s objectives, potential benefits, and procedures. Those who agreed to participate gave written informed consent. Demographic and obstetric data were then obtained using the structured interview questionnaire. For the study group, self-efficacy was assessed at recruitment using the ACSEI to support individualized educational emphasis. For the control group, baseline self-efficacy was not collected because recruitment occurred at labor admission; therefore, only intrapartum self-efficacy assessments were available for between-group comparisons. Anxiety and pain intensity were not assessed at baseline, as these are labor-specific outcomes. The control group was recruited from the labor unit at the time of admission, following similar consent and baseline data collection procedures, but without pre-intervention self-efficacy testing.

### Intervention phase: nursing-led childbirth preparedness sessions

The intervention consisted of two structured, nurse-led sessions, each lasting approximately 45 minutes, delivered between the 32^nd^ and 34^th^ weeks of gestation. To enhance learning and interaction, sessions were conducted in small groups of no more than five Primiparous women. The sessions combined didactic instruction, interactive discussions, demonstrations, and re-demonstrations, supported by audiovisual materials and the distribution of a culturally tailored Arabic-language booklet.

- **Session 1 (Educational Content):** This session began at the same day of recruitment of primiparous women, focused on providing foundational knowledge about childbirth. Topics included preparation of the labor bag, recognition of true versus false labor pains, identification of early signs of labor and a detailed explanation of the stages of labor. Emphasis was placed on what primiparous women could expect physiologically and emotionally during each stage. The session was delivered using illustrated PowerPoint slides and interactive discussions, encouraging Primiparous women to share their concerns and clarify misconceptions. Primiparous women was informed about the date and time of the second session according to date of follow-up for them.
- **Session 2 (Skill Development and Coping Strategies):** The second session was introduced the next week of the first session, emphasized practical skills aimed at enhancing primiparous women’s confidence in managing labor-related anxiety and pain. Primiparous women were taught various movement and position-changing techniques to facilitate labor progress and comfort, including walking, side-lying, squatting, and the use of upright positions. Breathing techniques were demonstrated and practiced, tailored to each phase of labor: slow breathing during early labor, light patterned breathing during the active phase, cleansing breaths at the onset and conclusion of contractions, “hee-hee-hoo” breathing for the transition phase, and pushing breaths during the second stage of labor. Role-playing and guided demonstrations were used to reinforce mastery. Emotional support was integrated throughout the sessions, allowing Primiparous women to express fears and receive reassurance from the nurse facilitator and peers.

At the conclusion of the second session, Primiparous women in the study group received a supportive Arabic-language booklet developed by the researcher. The booklet summarized all educational content, included photographs demonstrating positions and breathing techniques, and incorporated links to short instructional videos for home review. Primiparous women were encouraged to review the booklet with family members to ensure social support during childbirth.

### Follow-up phase

Following the sessions, primiparous women in the study group were contacted by telephone weekly to reinforce key concepts and provide an opportunity for questions. Primiparous women were asked to notify the investigator when the onset of labor occurred to allow timely follow-up at the labor unit.

### Evaluation phase

Primiparous women in both study and control groups were assessed during labor using standardized tools.

- **Self-efficacy** was assessed with the ACSEI during the active phase (first-stage items) and second stage of labor (second-stage items).
- **Anxiety level** was measured twice using the Anxiety Assessment Scale for Pregnant women in Labor (AASPWL): once at admission and once during the active phase before transition.
- **Pain intensity level** was measured hourly during the first stage of labor using the Numerical Pain Rating Scale (NRS). Pain intensity was assessed using the NRS at standardized time points (approximately every hour) during the first stage of labor, aligned with routine maternal observations in the unit. The same scoring instructions were used for all Primiparous women. Because the intervention involved antenatal education, blinding Primiparous women was not possible; however, NRS ratings were self-reported by the woman, which reduces observer influence. Where feasible, the nurse collecting NRS and anxiety scores was not involved in delivering the antenatal sessions.
- **Maternal assessments** were monitored through clinical observation and labor records, including total labor duration, progression, and maternal complications.

Data collection was completed at the end of the second stage of labor for both groups. The investigator ensured a supportive presence, minimizing interference with routine clinical care while systematically recording assessments.

### Data analysis

Data was analyzed using the Statistical Package for the Social Sciences (SPSS, version 25). Descriptive statistics, including means, standard deviations, and frequencies, summarized participant characteristics and outcome measures. Independent t-tests compared mean scores between groups, while chi-square tests assessed categorical variables. Parametric inferential statistics (T-test & Chi-square) was used to examine the differences between the study and control group. The level of significance will be *p*- value < 0. 05.

## Results

Table 1 presents distribution of primiparous women in both groups according to their demographic data. Across all examined demographic variables there were no statistically significant differences between the study and control groups which denote homogeneity between groups. the highest percentage of primiparous women in both groups was fill in the age category 18– less than 23 years (54.3% in the study group & 57.1% in the control group), the age ranged between (18–35) years, the highest percentage in both groups had preparatory education (35.7% in the study group & 38.6% in the control group), most of them were housewives (85.7% in the study group & 80.0% in the control group), and more than half of primiparous women in the study group were live in rural area (51.4%), versus 45.7% in the control group. For monthly income, most of primiparous women in both groups had inadequate income (71.4% in the study group; 68.6% in the control group).

Table 2 The results demonstrate statistically significant differences in Childbirth Self-Efficacy Inventory scores between primiparous women in the study and control groups during the first and second stages of labor. Across all domains of the Arabic Childbirth Self-Efficacy Inventory (ACSEI), the study group demonstrated consistently higher mean scores than the control group. During the first stage of labor, both outcome expectancy and self-efficacy expectancy were higher among primiparous women who attended the childbirth preparedness sessions, with these differences becoming more pronounced during the second stage. When scores from both stages were combined, the study group achieved significantly higher total outcome expectancy, total self-efficacy expectancy, and overall ACSEI scores compared with the control group (all *p* < 0.001), indicating improved childbirth self-efficacy among primiparous women who attended the childbirth preparedness sessions.

**Table 2:** Distribution of primiparous women in both groups according to their Mean scores of child-birth self-efficacy during labor

Measure	Study group (n = 70) Mean ± SD	Control group (n = 70) Mean ± SD	t-test	p-value
**Outcome Expectancy (OE)**				
(OE) 1st stage	54.31 ± 2.65	51.06 ± 2.59	7.34	< 0.001
(OE) 2nd stage	56.84 ± 2.27	50.06 ± 2.60	16.44	< 0.001
**Total Outcome Expectancy** (OE1 + OE2)	**111.15 ± 3.49**	**101.12 ± 3.67**	**16.57**	** < 0.001**
**Self-Efficacy Expectancy (SE)**				
(SE) 1st stage	55.76 ± 2.36	50.50 ± 2.54	12.69	< 0.001
(SE) 2nd stage	59.16 ± 2.64	48.63 ± 3.07	21.76	< 0.001
**Total Self-Efficacy Expectancy** (SE1 + SE2)	**114.92 ± 3.54**	**99.13 ± 3.98**	**24.80**	** < 0.001**
**Total ACSEI** (Total OE + Total SE)	**226.07 ± 7.12**	**200.25 ± 5.96**	**23.27**	** < 0.001**

**Note:** Outcome Expectancy (OE) and Self-Efficacy Expectancy (SE) scores were calculated by summing first-stage (15 items) and second stage (16 items) responses (31 items total; score range 31–310). Higher scores indicate greater childbirth self-efficacy

Table 3 shows that, highly statistically significant differences between groups regarding levels of child-birth self-efficacy during labor. The highest percentage of primiparous women in the study group were having high self-efficacy (44.3%), VS (22.9%) in the control group.

**Table 3:** Distribution of the primiparous women in both groups according to child-birth self-efficacy levels during labor

Category	Study Group (n = 70)	Control Group (n = 70)	χ^2^	p-value
Low	12 (17.1%)	39 (55.7%)	22.51	< 0.001
Moderate	27 (38.6%)	15 (21.4%)		
High	31 (44.3%)	16 (22.9%)		

**Cut of values**: Total score of childbirth self-efficacy is categorized as low (31–103), moderate (104–206), or high (207–310)

Table 4 shows a statistically significant difference between the study and control groups regarding labor anxiety levels. The study group demonstrated a higher percentage of primiparous women in the low anxiety level (51.4%) compared with the control group (22.9%), whereas higher and severe anxiety levels were more prevalent among primiparous women in the control group.

**Table 4:** Distribution of the primiparous women in both groups according to anxiety levels

levels of Anxiety Assessment Scale for Pregnant Primiparous women in Labor (AASPWL)	Study (n = 70)	Control (n = 70)	Chi-Square χ^2^	p-value
Low (9–18)	36 (51.4%)	16 (22.9%)	**14.53**	**0.002**
Moderate (19–27)	22 (31.4%)	26 (37.1%)		
High (28–36)	8 (11.4%)	17 (24.3%)		
Severe (37–45)	4 (5.7%)	11 (15.7%)		

Table 5 shows labor pain intensity categories in latent and active acceleration phases. No statistically significant difference was detected in the latent phase (*p* = 0.63). In the active acceleration phase, the distribution differed between groups (*p* = 0.047), indicating a statistically significant but borderline difference; therefore, this finding should be interpreted cautiously.

**Table 5:** Distribution of the primiparous women in both groups according to labor pain intensity levels during first stage of labor

Variable	Pain Intensity Levels	Study (n = 70)	Control (n = 70)	Chi-Square χ^2^	p-value
latent phase (NRS)	Mild (1–3)	10 (14.3%)	8 (11.4%)	0.93	0.63
	Moderate (4–6)	50 (71.4%)	48 (68.6%)		
	Severe (7–10)	10 (14.3%)	14 (20.0%)		
Active Acceleration phase (NRS)	Mild (1–3)	3 (4.3%)	1 (1.4%)	6.10	0.047
	Moderate (4–6)	32 (45.7%)	20 (28.6%)		
	Severe (7–10)	35(50.0%)	49 (70.0%)		

*Cut-offs used: NRS: Mild (1–3), Moderate (4–6), Severe (7–10)*

Table 6 shows that primiparous women in the study group consistently reported lower mean labor pain scores than those in the control group across latent and active acceleration phase of first stage of labor. These differences were statistically significant.

**Table 6:** Comparison of mean labor pain scores during latent and active acceleration phase of first stage of labor between primiparous women in study and control groups

Labor Phase	Study Group		Control Group		t-test	p-value
	Mean	SD	Mean	SD		
Latent phase	4.66	0.68	4.96	0.82	−2.35	0.020
Active acceleration phase	6.07	0.80	7.14	0.98	−7.06	< 0.001

Table 7 shows that first and second stages durations were consistently shorter in the study group. The study group had shorter first-stage (8.85 ± 1.89) while the control group had longer first-stage (10.17 ± 1.94 hours). The differences in first stage and second stage were all statistically significant (*p* < 0.001).

**Table 7:** Comparison of mean first and second stage duration between primiparous women in study and control groups

Measure	Study (n = 70)	Control (n = 70)	t- test	*p***-value**
First stage duration (hours)	8.85 ± 1.89	10.17 ± 1.94	−4.01	< 0.001
Second stage duration (min)	63.9 ± 22.6	89.6 ± 26.3	−6.01	< 0.001

Table 8 The findings reveal that vaginal delivery with episiotomy was the most common mode in both groups but occurred more frequently in the study group (77.1%) than in the control group (55.7%). Conversely, cesarean section rates were lower in the study group (17.1% vs. 34.3%). Overall, a statistically significant difference in mode of delivery was observed between the two groups (χ^2^ = 7.24, *p* = 0.027).

**Table 8:** Distribution of primiparous women in study and control groups according to mode of delivery

Mode of Delivery	Study (n = 70)	Control (n = 70)	Chi-Square χ^2^	p-value
Vaginal delivery (with episiotomy)	54 (77.1%)	39 (55.7%)	**7.24**	**0.027**
Instrumental delivery	4 (5.7%)	7 (10.0%)		
Cesarean section	12 (17.1%)	24 (34.3%)		

## Discussion

This quasi-experimental study’s findings provide support for all the stated hypotheses.

Findings of the current study strongly support H1: which hypothesized that, Primiparous women who attend a childbirth preparedness session will have a higher level of self-efficacy during labor than those who don’t attend a childbirth preparedness session. The study group achieved significantly higher scores across all components of the Arabic Childbirth Self-Efficacy Inventory, including outcome expectancy, self-efficacy expectancy, and total ACSEI scores during both the first and second stages of labor. In addition, categorical analysis revealed that a substantially higher percentage of primiparous women in the study group were classified as having high self-efficacy, whereas most primiparous women in the control group remained within the low self-efficacy category. This pattern indicates not only statistical improvement but also a clinically meaningful shift in primiparous women’s perceived ability to cope with labor.

The pattern of findings is particularly relevant for resource-constrained maternity settings where women may have limited access to structured antenatal education. In such contexts, nurse-led sessions can provide a feasible mechanism to strengthen coping confidence and reduce uncertainty during childbirth. The observed improvements in self-efficacy and associated outcomes are consistent with prior evidence suggesting that structured, skills-based antenatal education is more effective than information-only approaches, especially when culturally tailored materials and follow-up reinforcement are included [19].

These findings are consistent with Bandura’s self-efficacy theory, which posits that mastery experiences, verbal persuasion, and cognitive preparation enhance perceived capability to manage challenging situations [20]. Lowe’s application of this theory to childbirth emphasizes that antenatal education can directly influence women’s confidence in coping with labor. Empirical evidence strongly supports this mechanism. [6]. Rahimparvar, et al. conducted a randomized controlled trial in Hong Kong and found that women who participated in a structured self-efficacy–enhancing antenatal program reported significantly higher childbirth self-efficacy and lower perceived helplessness during labor compared with controls [21].

Differences across studies may reflect variability in intervention “dose” and content. Programs that are brief, lecture-based, or focused mainly on information dissemination may improve knowledge without meaningfully changing coping confidence or fear-related arousal. In contrast, interventions that include skills rehearsal (breathing/relaxation, positioning, cognitive reframing), opportunities for interaction, and reinforcement (e.g., follow-up calls) are more likely to translate into measurable reductions in anxiety and pain and improved labor progress. Cultural context may also shape effects, as norms regarding childbirth fear, family support, and expectations about pain can influence how women interpret labor sensations and apply coping skills [22].

Similarly, Brixval et al. conducted RCT for assessing the effect of structured Antenatal education in small classes versus auditorium-based lectures on increasing childbirth self-efficacy, they found that women in the intervention group reported statistically significant higher levels of confidence in their ability to cope during labor compared to the control group. Likewise, the intervention had a positive effect on the women’s confidence in own ability to handle the birth process. [23]. Further support comes from Hildingsson et al., who reported that women attending antenatal preparation classes experienced greater confidence, perceived control, and emotional readiness for childbirth [24].

In contrast, some studies have reported weaker or non-significant effects, particularly when antenatal education is brief, unstructured, or purely informational. For example, Kaya (2025) examined the effectiveness of antenatal education programs delivered to primiparous women and found that classes emphasizing general information about pregnancy and childbirth, without structured training in coping strategies (such as breathing techniques, relaxation exercises, or cognitive reframing), produced limited improvements in psychosocial outcomes. Although Primiparous women in this study demonstrated increased factual knowledge, there were no significant reductions in anxiety levels or meaningful gains in perceived control during labor compared with women receiving standard care. [25]. These findings suggest that antenatal education alone is insufficient unless it actively engages women in skill-based coping practices, underscoring that both content depth and interactive delivery style are critical determinants of intervention effectiveness. Overall, the present findings strongly confirm H1, highlighting the effectiveness of structured childbirth preparedness sessions in enhancing childbirth self-efficacy.

The findings of the current study support H2: which hypothesized that, Primiparous women who attend a childbirth preparedness session will report lower anxiety level than those who don’t attend a childbirth preparedness session. In the present study, the significant reduction in anxiety levels suggests that the childbirth preparedness sessions were sufficiently comprehensive and contextually relevant to influence emotional outcomes. Thus, H2 is supported.

The study group included a higher percentage of primiparous women in the low anxiety category and fewer women in the high and severe anxiety categories, with the overall difference in anxiety distribution between groups reaching statistical significance. labor anxiety is linked to increased sympathetic activity, elevated catecholamines, muscle tension, and heightened pain perception. Childbirth preparedness sessions reduced this stress response by improving understanding of labor, decreasing uncertainty, and strengthening coping strategies such as breathing and relaxation, thereby promoting better emotional regulation and lower anxiety during labor.

These findings are supported by prior research indicating that childbirth-focused education and psychological preparation can reduce anxiety. Hosseini et al. conducted a controlled intervention among women with fear of childbirth in Finland and found significant reductions in childbirth-related anxiety following structured counseling and education [26]. Similarly, O’Connell et al. reported that antenatal education programs improved emotional preparedness and reduced childbirth fear, particularly when education included coping strategies and reassurance [27].

On the other hand, literature also includes studies that did not observe significant reductions in anxiety following antenatal education. For instance, Hadaro et al. reported in their study that although antenatal educational interventions were effective in enhancing women’s confidence and perceived preparedness for childbirth, their impact on anxiety reduction was inconsistent. Anxiety outcomes were strongly shaped by pre-existing fear of childbirth, prior expectations, and sociocultural beliefs surrounding labor and pain, which often persisted despite education. Also authors emphasized that without targeted psychological components addressing fear and emotional regulation, antenatal programs may improve self-efficacy while leaving underlying anxiety largely unchanged [28].

Likewise, Çankaya et al., in a quasi-experimental study of pregnant women attending routine antenatal education programs found that routine antenatal education had limited impact on severe childbirth fear unless supplemented with targeted psychological interventions. These mixed findings suggest that anxiety is a complex construct influenced by personality traits, prior experiences, and cultural context [29].

Findings of the current study provide strong support for H3, which hypothesized that, Primiparous women who attend a childbirth preparedness session will report lower labor pain level than those who don’t attend a childbirth preparedness session. Although moderate pain predominated during the latent phase in both groups, primiparous women in the study group experienced significantly less severe pain during the active acceleration phase of the first stage of labor. Mean pain scores further confirmed significantly lower pain intensity in the study group during both latent and active phases. suggesting that childbirth preparedness sessions enhanced women’s ability to interpret and cope with labor sensations more effectively. Improved understanding of labor physiology and the use of coping strategies such as controlled breathing techniques may have reduced fear-related tension and modulated labor pain perception, resulting in lower reported pain intensity.

These findings align with the fear–tension–pain model, which proposes that fear and anxiety increase muscle tension and amplify pain perception, whereas education and coping strategies can interrupt this cycle. Empirical support for this model is robust. Vaid et al. [30], conducted a randomized controlled trial to examine the effectiveness of supportive care combined with relaxation techniques during labor on maternal pain and anxiety. The intervention included continuous emotional support, guided breathing, and relaxation strategies provided during labor, and the authors reported that women in the intervention group experienced significantly lower pain intensity and reduced anxiety levels compared with those receiving routine care.

Similarly, Alizadeh-Dibazari et al. [17] conducted quasi-experimental study to evaluate the effect of structured antenatal childbirth education classes on maternal experiences during labor [17]. The program focused on childbirth knowledge, breathing techniques, relaxation, and coping strategies. Their findings showed that women who attended the structured antenatal classes reported lower perceived labor pain and higher childbirth satisfaction compared with women who received routine antenatal care.

Conversely, some studies have reported limited or inconsistent effects of antenatal education on pain perception. Hogan et al. [31] conducted a systematic review “Antenatal education programs and their impact on labor pain and birth outcomes” reported that antenatal education showed inconsistent effects on labor pain intensity, with more consistent benefits observed only when programs incorporated active coping skills, relaxation techniques, or continuous support [31]. These findings indicate that antenatal education alone may be insufficient to substantially influence pain outcomes without interactive and skills-based components [32]. These discrepancies suggest that pain reduction depends on active skills training, such as breathing, relaxation, and cognitive reframing, rather than information alone.

The findings of the current study clearly support H4, which hypothesized that, Primiparous women who attend a childbirth preparedness session will have shorter labor duration than those who don’t attend a childbirth preparedness session. Primiparous women in the study group experienced significantly shorter first- and second-stage labor durations compared with the control group, with differences that were both statistically and clinically meaningful. Improved preparedness reduced fear-related sympathetic activation and catecholamine release, which are known to inhibit uterine contractility, increase maternal muscle tension, and contribute to dysfunctional or prolonged labor. By strengthening coping skills as breathing techniques, the sessions helped in promoting better emotional regulation, more efficient maternal pushing, and improved coordination between uterine contractions and maternal effort, thereby facilitating labor progress. Additionally, the mode of delivery differed significantly between groups, with higher rates of vaginal delivery and lower cesarean section rates among primiparous women who attended the preparedness sessions. The significantly higher rate of vaginal delivery and lower cesarean section rate in the study group further suggests that enhanced readiness and reduced stress contributed to fewer labor complications and less need for operative intervention.

These findings are consistent with physiological and psychological models linking reduced stress and enhanced self-efficacy to more efficient labor. Çankaya & Can [33], demonstrated that emotional support and reduced fear during labor were associated with shorter labor duration and fewer obstetric interventions. Supporting evidence also comes from Hildingsson et al. [34], who reported that fear of childbirth was associated with prolonged labor and increased cesarean section rates [35]. In contrast, some large observational studies, such as Vogel et al., found that labor duration is multifactorial and not solely dependent on psychological preparedness [36]. Nonetheless, the convergence of shorter labor duration and more favorable delivery outcomes in the present study provides strong evidence in support of H4.

### Implications of the study

The findings of this study hold several important implications for nursing practice, education, and policy. First, the significant improvement in childbirth self-efficacy underscores the effectiveness of structured, nurse-led antenatal education as a low-cost, scalable strategy to support Primiparous women during labor. Integrating such programs into routine antenatal care can enhance maternal confidence, reduce reliance on pharmacological interventions, and promote positive birthing experiences. Second, the demonstrated reductions in anxiety, pain, and labor duration highlight the potential of nursing interventions to influence both psychological and physiological outcomes, thereby reinforcing the holistic role of nurses in maternal health care. Third, the results provide evidence to inform policymakers and healthcare administrators about the value of prioritizing antenatal preparedness programs, especially in resource-constrained settings such as Egypt. Finally, the study supports the incorporation of self-efficacy–based frameworks into nursing curricula to strengthen the future workforce’s capacity to deliver evidence-based maternal education.

### Limitations

1. The quasi-experimental, non-randomized post-test-only design may limit the strength of causal inferences and does not fully control for potential confounding variables.
2. The study was conducted in a single tertiary university hospital using purposive sampling, which may limit the transferability of the findings to other maternity care contexts, such as rural settings, private hospitals, or primary healthcare facilities.
3. The sample was restricted to low-risk primiparous women who were able to read and write Arabic, which may limit the applicability of the findings to women with high-risk pregnancies or lower literacy levels.
4. These contextual and methodological characteristics should be considered when interpreting the findings, and future studies using multi-center randomized or pragmatic trial designs are warranted to enhance generalizability across diverse maternity care settings.

### Recommendations of the study

Based on the findings of the current study, the following recommendations are proposed:

1. Raise pregnant women’s awareness regarding benefits of childbirth preparedness sessions.
2. Integrate structured childbirth preparation programs into routine antenatal care for pregnant women, especially primiparous women.
3. Develop appropriate educational materials tailored to women’s needs and literacy levels to support learning and skill acquisition during pregnancy.
4. Expand childbirth preparedness programs to include husband or support persons in further studies.
5. For research, it’s recommended to conduct a Qualitative Study ‘Women’s Perspectives on Comprehensive Childbirth Preparedness’.

## Conclusion of the study

In this quasi-experimental study, participation in structured nurse-led childbirth preparedness sessions was associated with higher childbirth self-efficacy and more favorable intrapartum outcomes, including lower anxiety and pain, shorter labor stages, and a lower proportion of cesarean births. Because the design was non-randomized and baseline psychosocial equivalence could not be fully established, causality cannot be confirmed. Future multi-center randomized trials and pragmatic implementation studies are recommended to verify effectiveness and inform broader integration into antenatal care.

## Data Availability

The datasets generated and/or analyzed during the current study are not publicly available due to institutional data protection policies but are available from the corresponding author on reasonable request.
